# Supplementary material for: Medication multiple experiences of elderly patient with multiple chronic condition: A qualitative meta-synthesis
Source: PLoS One. 2025 Sep 9;20(9):e0331753. doi: 10.1371/journal.pone.0331753 (PMC12419632; doi:10.1371/journal.pone.0331753)
Supplement: S1 File — (DOCX) [file pone.0331753.s001.docx]

**S1 File.**. **Search strategy.**

Take the PubMed database retrieval strategy as an example

| Date |  | Keywords | Search results | |
| --- | --- | --- | --- | --- |
| 30/10/2024 | #1 Aged [MeSH Terms] OR elderly [Title/Abstract] OR elderly [Title/Abstract] OR old adults [Title/Abstract]  #2 Multiple Chronic Conditions [MeSH Terms] OR Multiple Chronic Health Conditions [Title/Abstract] OR Multiple Chronic Medical Conditions [Title/Abstract] OR Multiple Chronic Illnesses [Title/Abstract] OR Multiple Chronic Illnesses [Title/Abstract] OR comorbidity[Title/Abstract]  #3 polypharmacy[MeSH Terms] OR polymedication [Title/Abstract] OR multiple medication[Title/Abstract] OR multiple drug[Title/Abstract] OR Medicine drug[Title/Abstract]  #4 medication therapy management[MeSH Terms] OR drug therapy management[Title/Abstract] OR medicines management[Title/Abstract] OR medication management, medicines management[Title/Abstract]  #5qualitative research[MeSH Terms] OR ethnographic research[Title/Abstract] OR narrative research[Title/Abstract] OR phenomenology[Title/Abstract] OR interview[Title/Abstract] OR experience[Title/Abstract] OR opinion[Title/Abstract]  #6 #1AND#2AND#3AND#4AND5 | | 1315 |  |
