# Supplementary figures and images for: Medication multiple experiences of elderly patient with multiple chronic condition: A qualitative meta-synthesis
Source: PLoS One. 2025 Sep 9;20(9):e0331753. doi: 10.1371/journal.pone.0331753 (PMC12419632; doi:10.1371/journal.pone.0331753)

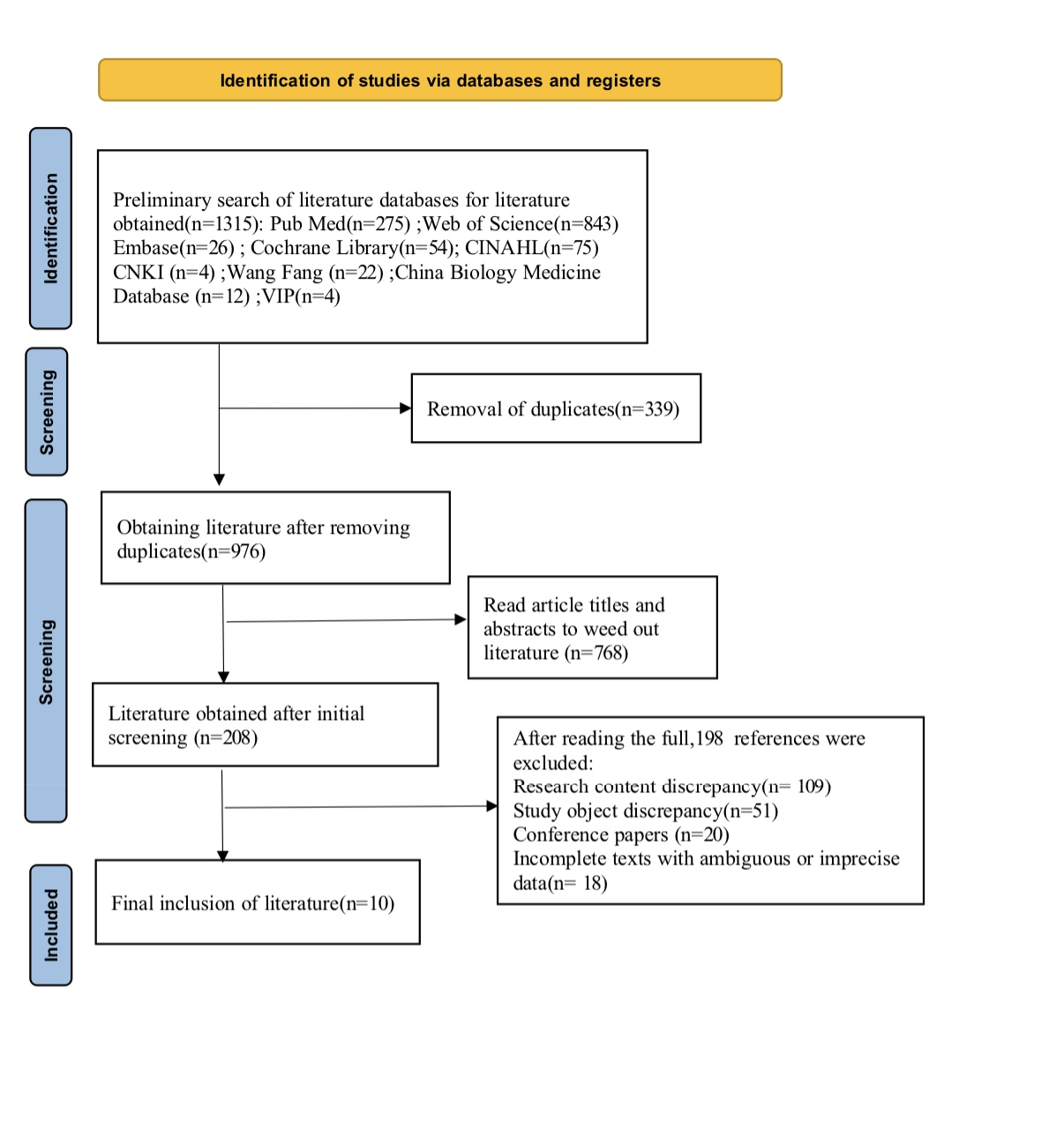

Supplement: S1 Fig — (TIF) [file pone.0331753.s002.tif]
